# Bibliometric analysis of sepsis and gut microbiota: Trends from 2014 to 2024

**DOI:** 10.3389/fmicb.2025.1598443

**Published:** 2025-06-19

**Authors:** Runze Zhang, Chenxing Huo, Chenming He, Jielian Luo, Yunan Shan, Jiamin Lu, Jirong Zhang, Wen Zhang, Bangjiang Fang

**Affiliations:** ^1^Department of Emergency, Longhua Hospital Affiliated to Shanghai University of Traditional Chinese Medicine, Shanghai, China; ^2^School of Traditional Chinese Medicine, Hubei University of Chinese Medicine, Wuhan, China; ^3^Institute of Emergency and Critical Care Medicine, Shanghai University of Traditional Chinese Medicine, Shanghai, China

**Keywords:** bibliometric, CiteSpace, gut microbiota, sepsis, VOSviewer

## Abstract

**Background:**

Sepsis is a life-threatening condition characterized by organ dysfunction resulting from a dysregulated host response to infection, with pathological mechanisms closely linked to imbalances in the intestinal flora. While the gut microbiota influences sepsis progression through metabolic and immune regulation, systematic analyses of research trends remain limited. This bibliometric study comprehensively evaluates the sepsis and gut microbiota field from 2014 to 2024.

**Methods:**

We analyzed 944 English-language articles from the Web of Science (WOS) Core Collection using CiteSpace, VOSviewer, and other software tools. The bibliometric assessments included analysis of publication trends, collaborative networks, geographic distributions, journal impact, and keyword clustering.

**Results:**

A total of 944 publications included contributions from 5,901 authors across 69 countries. These works were published in 405 journals, and cited a total of 45,932 references. The focus of research has transitioned from early topics such as “necrotizing enterocolitis” and “premature infants” to more recent interests on “short-chain fatty acids” (SCFAs) and “*Candida albicans*.” Futhermore, emerging topics include “sepsis-associated encephalopathy,” with microbiology and critical care medicine identified as interdisciplinary core fields.

**Conclusion:**

The therapeutic potential of the gut microbiota in sepsis is increasingly recognized. Future research should prioritize microbial-targeted therapies, immune-barrier-metabolic network regulation, and integrated traditional Chinese–Western medicine approaches.

## 1 Introduction

Sepsis is defined as a life-threatening condition characterized by organ dysfunction resulting from a dysregulated host response to infection (Wang and Liu, 2023). Sepsis is not only a major public health problem but also a common cause of death in hospitalized patients. To raise awareness of the disease, the World Health Organization designated September 13 as World Sepsis Day in 2012 (Schlapbach et al., 2020).

The gut microbiota is a group of microorganisms that reside in the human gut and depend on each other for a long time (Gomaa, 2020). These microbes utilize macronutrients from the diet and secretions from intestinal epithelial cells as substrates, undergo a series of metabolic reactions in the intestine, and produce a large number of metabolites that can be either beneficial or harmful to the human body (Feng et al., 2018). Microbes play an important role in maintaining the function of the intestinal mucosal barrier and regulating the function of the innate and adaptive immune systems. The immune system is closely related to the location and composition of the intestinal microbiota. Sepsis seriously affects the composition of the intestinal flora, which may contribute to the occurrence and development of organ failure. The uncontrolled inflammatory response in sepsis can not only cause serious damage to the gut itself (Park et al., 2024) but can also lead to severe flora disorder and disrupt the production of intestinal flora metabolites (Festi et al., 2014).

Based on the Web of Science (WOS) database, this study comprehensively analyzed the current status of research on intestinal flora in sepsis using bibliometric methods and aimed to reveal research trends in this field and predict the future possible research hotspots.

## 2 Materials and methods

### 2.1 Data sources

We selected the Science Citation Index Expanded (SCIEXPANDED) from the Web of Science Core Collection for bibliometric analysis. The search terms were as follows: (TS = (“sepsis” OR “septicemia” OR “septic shock” OR “septicaemia”) AND TS = (“gut microbiota” OR “gut microbiome” OR “intestinal microbiota” OR “intestinal microbiomegastrointestinal microbiota” OR “gastrointestinal microbiome” OR “gut flora” OR “intestinal flora”)). The time frame was from 1 January 2014 to 31 December 2024. To ensure reliability, the data in this study were screened independently by two authors according to the inclusion and exclusion criteria. In case of disagreement, a third author reviewed and confirmed the selection to ensure that the included literature met the criteria for the report.

### 2.2 Data collection and cleaning

Overall, 995 articles were initially retrieved from the Web of Science database. The exclusion criteria were as follows: (1) non-article and review articles, (2) retracted and duplicate articles, (3) non-English articles, and (4) other non-representative items. After screening, 944 English-language articles were finally included for analysis. The included literature was exported in both “fully documented and cited references” and “plain text” formats. All data in this report were extracted on the same day, 4 January 2025, to avoid bias caused by daily updates to the database. A total of 944 articles were included.

**Figure 1:**
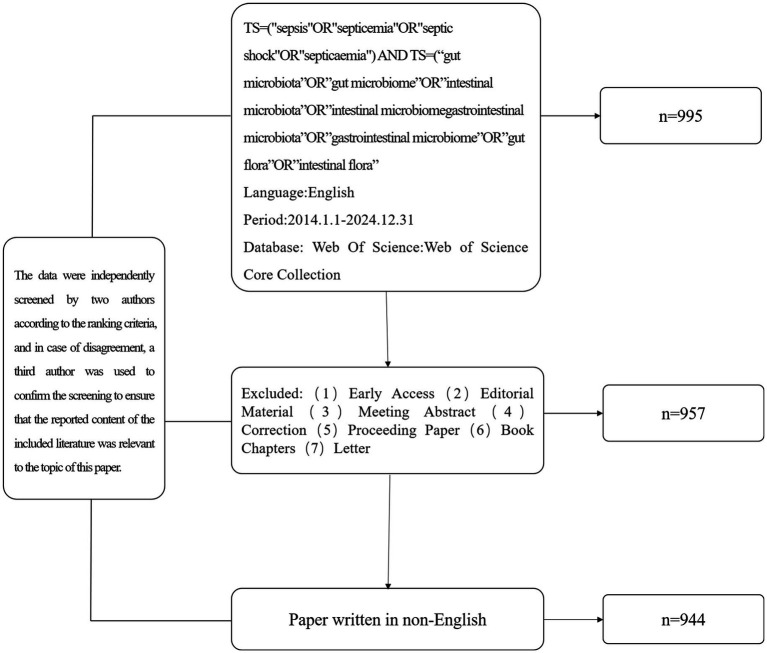
Flow chart of the literature screening.

The information extracted from the original data included the number of publications, citation frequencies, and details on countries and regions, publication years, institutions, authors, references, journals, and keywords. All data were obtained from an open-access database. Although the data might have been biased due to factors such as identical abbreviations used by different authors and multiple versions of cited literature, strict measures were taken to improve reliability. Multiple investigators reviewed the data at critical stages, merged synonymous terms, corrected spelling errors, and applied other methods to minimize potential inaccuracies (Figure 1).

### 2.3 Bibliometric analysis

This study used several software tools and platforms, including CiteSpace, VOSviewer, Pajck, Gephi, Tableau Public, and Charticulator.^1^ Among these, CiteSpace and VOSviewer are two important tools commonly used in bibliometric analysis. CiteSpace was developed by Chen (Chen, 2004). It is the most widely used bibliometric analysis software. VOSviewer was developed by Nees Jan van Eck et al (van Eck and Waltman, 2010), and it is mainly used for bibliometric network diagram analysis. In the study, visual analysis was conducted on the publishing country, publishing institution, author, journal, and keywords. Visualization charts were created for an in-depth analysis to summarize the current research status, hotspots, and emerging trends in the field of intestinal flora and sepsis. The analysis time slice in the CiteSpace software was set from January 2014 to December 2024, and the year was selected as “1.” The node screening method used was the g-index, and the k-value was set to 25 (Chen, 2017). Keywords were selected using the default settings, and the threshold values were kept at the system defaults.

## 3 Results

### 3.1 Analysis of the annually published articles

**Figure 2:**
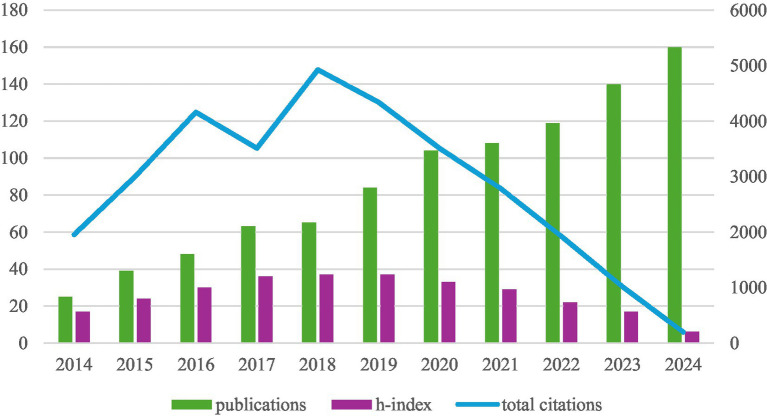
Annual publication volume, annual citation count, and the h-index of the literature related to sepsis and intestinal flora.

From 1 January 2014 to 31 December 2024, a total of 944 articles were retrieved. Figure 2 illustrates the annual number of publications, annual citations, and the h-index of the literature related to sepsis and gut microbiota. From the perspective of the number of articles published, there is a year-by-year increasing trend, suggesting that global researchers’ attention to this field continues to grow. Total citations showed a small fluctuation trend from 2016 to 2018 and have decreased year by year since 2018. The h-index showed an upward trend from 2014 to 1,018 and a downward trend from 2019 to 2024. The 944 articles were cited a total of 32,489 times, and the average citation frequency of each article was 34.42. The h-index was 90.

### 3.2 Author output analysis

By analyzing the authors of the studies, we can identify the representative scholars and core research forces in the research field. According to Price’s law (Price, 2019), the minimum number of articles published by core authors in this field is m ≈ 2.803. Therefore, authors who published more than three articles were considered core authors in this field. There were 219 core authors who published 900 articles in total, which was more than 50% of the total number of articles published and met the half standard proposed by Price. Therefore, it can be considered that a relatively stable group of core authors has formed in this field. Table 1 presents the top five authors with the highest number of publications in the field.

**Table 1:** The top five authors by number of publications.

Rank	Author	Documents	Citations	Average citation
1	Wiersinga, W. Joost	14	1948	139.14
2	Alverdy, John C.	12	657	54.75
3	Leelahavanichkul, Asada	11	354	32.18
4	Niemarkt, Hendrik J.	11	294	26.72
5	de Meij, Tim G. J.	11	243	22.09

The author analysis revealed that Wiersinga, W. Joost, who published the most articles in this field, ranked first with 16 articles. Professor W. Joost Wiersinga has deeply discussed the pathogenesis of sepsis, especially the host immune response to infection, and provided a theoretical basis for the immunomodulatory treatment of sepsis (Wiersinga and van der Poll, 2022). Professor John C. Alverdy is a pioneer in the research of gut microbiota and postoperative infections. He proposed an innovative strategy for the prevention of surgical site infections through targeted modulation of the gut microbiota, aiming to reduce reliance on traditional antibiotics (Hyoju et al., 2022). Asada Leelahavanichkul studied sepsis-induced multiple organ failure and specifically identified iron overload as an important cause of endogenous sepsis in patients with *β*-thalassemia (Visitchanakun et al., 2021). The author emphasizes the necessity of iron chelation therapy and intestinal protection strategies, providing experimental evidence for targeting intestinal barrier repair or regulating iron metabolism to reduce the risk of infection. Hendrik J. Niemarkt focused on the microbiome mechanisms of necrotizing enterocolitis (NEC) in preterm infants, aiming to establish an early warning model for NEC and facilitate clinical intervention (Deianova et al., 2023). Tim G. J. de Meij developed non-invasive diagnostic techniques based on volatile organic compounds (VOCs) (van Liere et al., 2023) to achieve rapid detection of intestinal inflammation and dysbiosis.

### 3.3 Journal analysis

Research on sepsis and gut flora is extensive, and the data from the WOS database showed that a total of 405 English-language journals worldwide have published scholarly articles on this topic. Table 2 lists the top 10 journals in terms of the number of articles published, and the high publication frequency suggests that current research is mainly focused on microbiology, immunology, and critical care. Among these journals, Frontiers in Microbiology has published the most articles, with a total of 34. PLOS One, Scientific Reports, Critical Care, and Pediatric Research have significantly higher citation counts than other journals, which indicates that clinical research tends to attract more attention.

**Table 2:** The top 10 journals in terms of the number of publications.

Rank	Source	Documents	Citations	Average citation
1	Frontiers in Microbiology	34	892	26.35
2	Frontiers in Immunology	33	931	28.21
3	Frontiers in Cellular and Infection Microbiology	25	296	11.84
4	Nutrients	24	586	24.41
5	PLoS One	22	1,077	48.95
6	Scientific Reports	20	1,087	54.35
7	Pediatric Research	18	847	47.05
8	Shock	17	407	23.94
9	International Journal of Molecular Sciences	15	285	19
10	Critical Care	15	1,049	69.93

**Figure 3:**
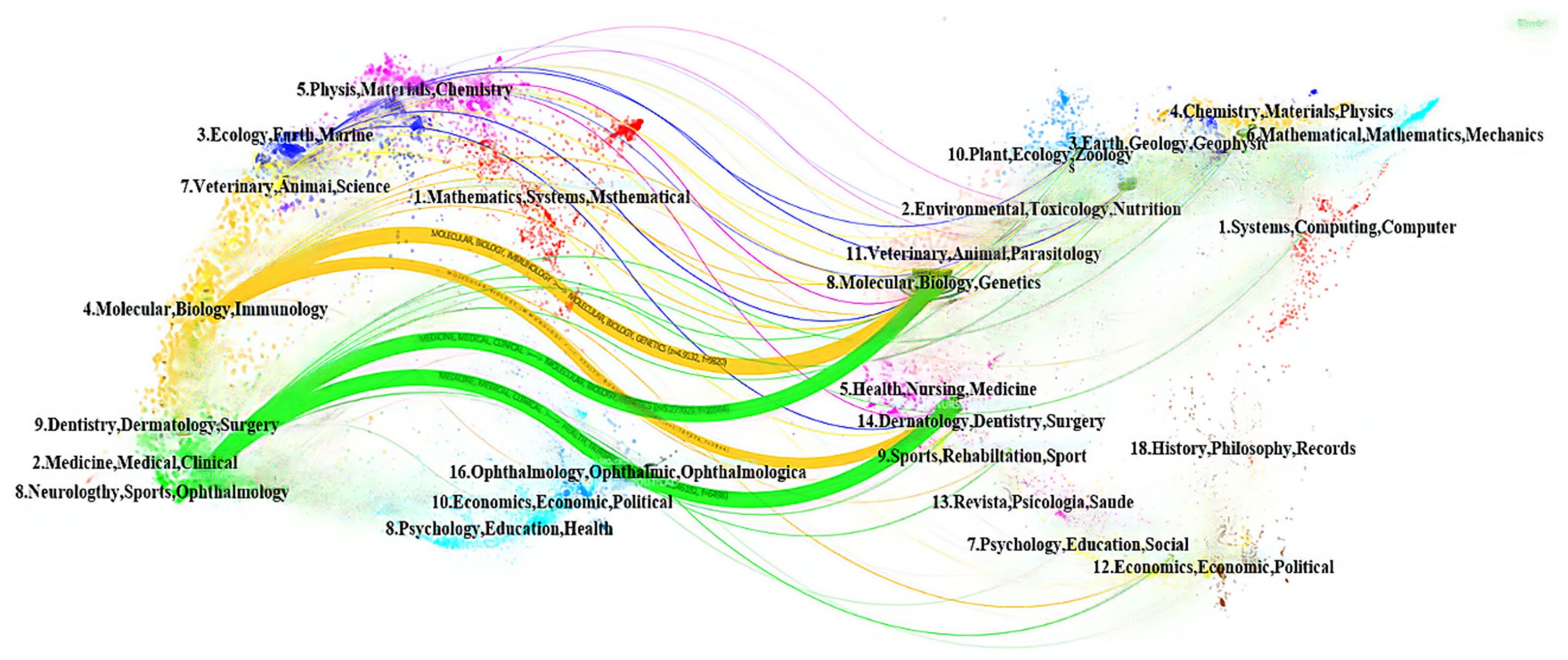
Visualization of the citation journal network, with colored paths indicating citation relationships.

Figure 3 presents a visualization of the journal clustering based on time-zone analysis. The overlay of the double graph reveals the macro-structural citation relationship between the knowledge frontier and the knowledge base. The citing journals are shown on the left, while the cited journals are shown on the right. The colored line paths indicate citation relationships, denoting knowledge citation trajectories and the flow of knowledge. As shown in the figure, there were four main citation paths that showed a cross-disciplinary development. They were as follows: “molecular, biology. Immunology→molecular, bioligy, genetics (z = 4.9132,*f* = 4,910), “molecular, biology, immunology→health, nursing, medicine(z = 1.787,*f* = 1972),” “medicien, medical, cinical→molecular, biology, genetics (z = 5.277029, *f* = 5,252),” and “medicine, medical, clinical→health, nursing, medicine (z = 2.146182, *f* = 3,249).” The *f*-value indicates the number of citations from the citing journal clusters to the cited journal clusters, and the z-value is a normalized measure of the f-value.

### 3.4 Analysis of institutional cooperation

**Figure 4:**
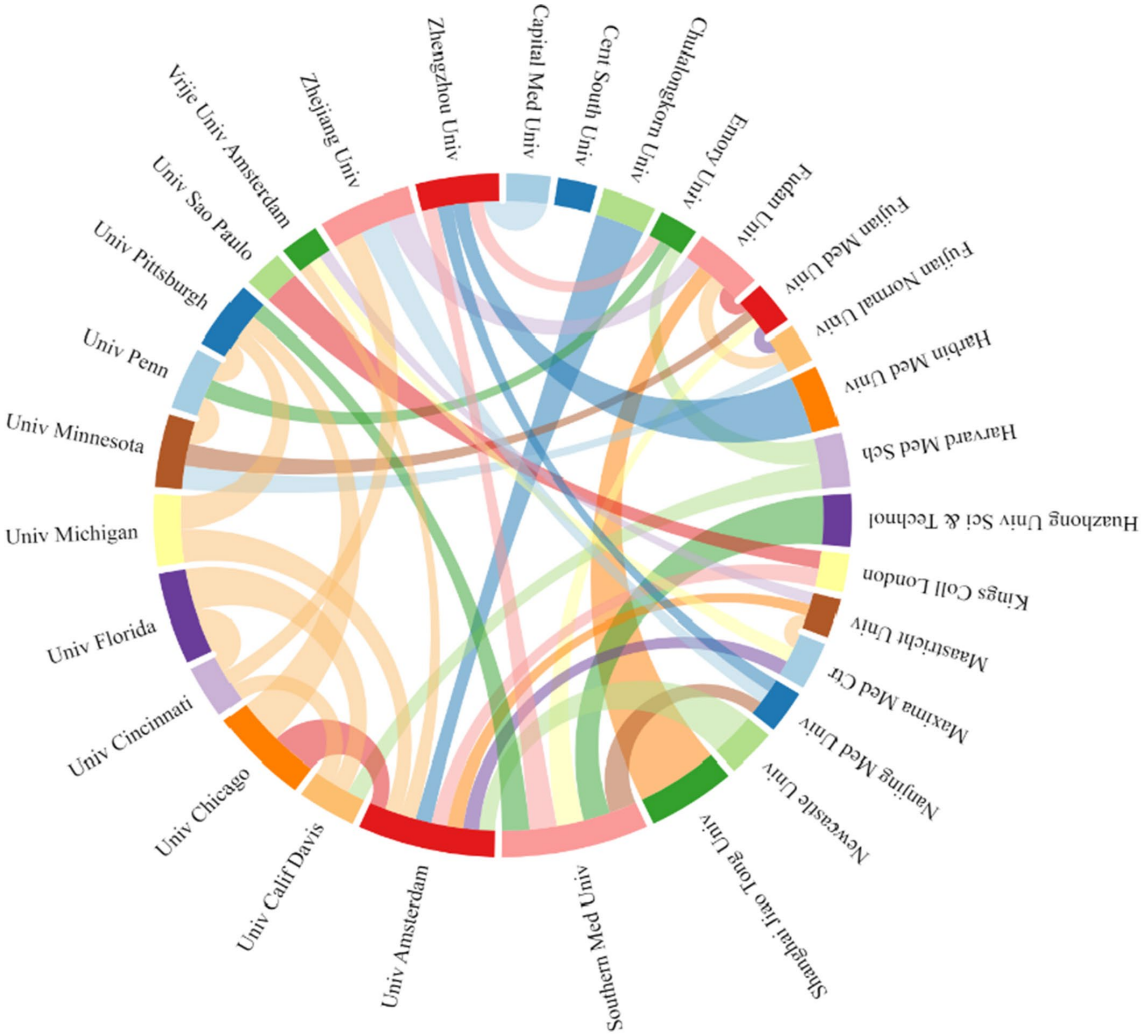
Institutional cooperation map.

A total of 1,539 institutions were included in the study, The top five institutions that published the most articles were Southern Medical University (*n* = 30), University of Amsterdam (*n* = 28), University of Chicago (*n* = 20), University of Florida (*n* = 19), and Zhejiang University (*n* = 19). Figure 4 shows that institutions with high productivity occupy core positions, and the intensity of cooperation among them is strong, which greatly affects inter-institutional cooperation. It is recommended to strengthen inter-agency cooperation and promote the breadth and depth of research development.

### 3.5 Analysis of the nationally published articles

**Figure 5:**
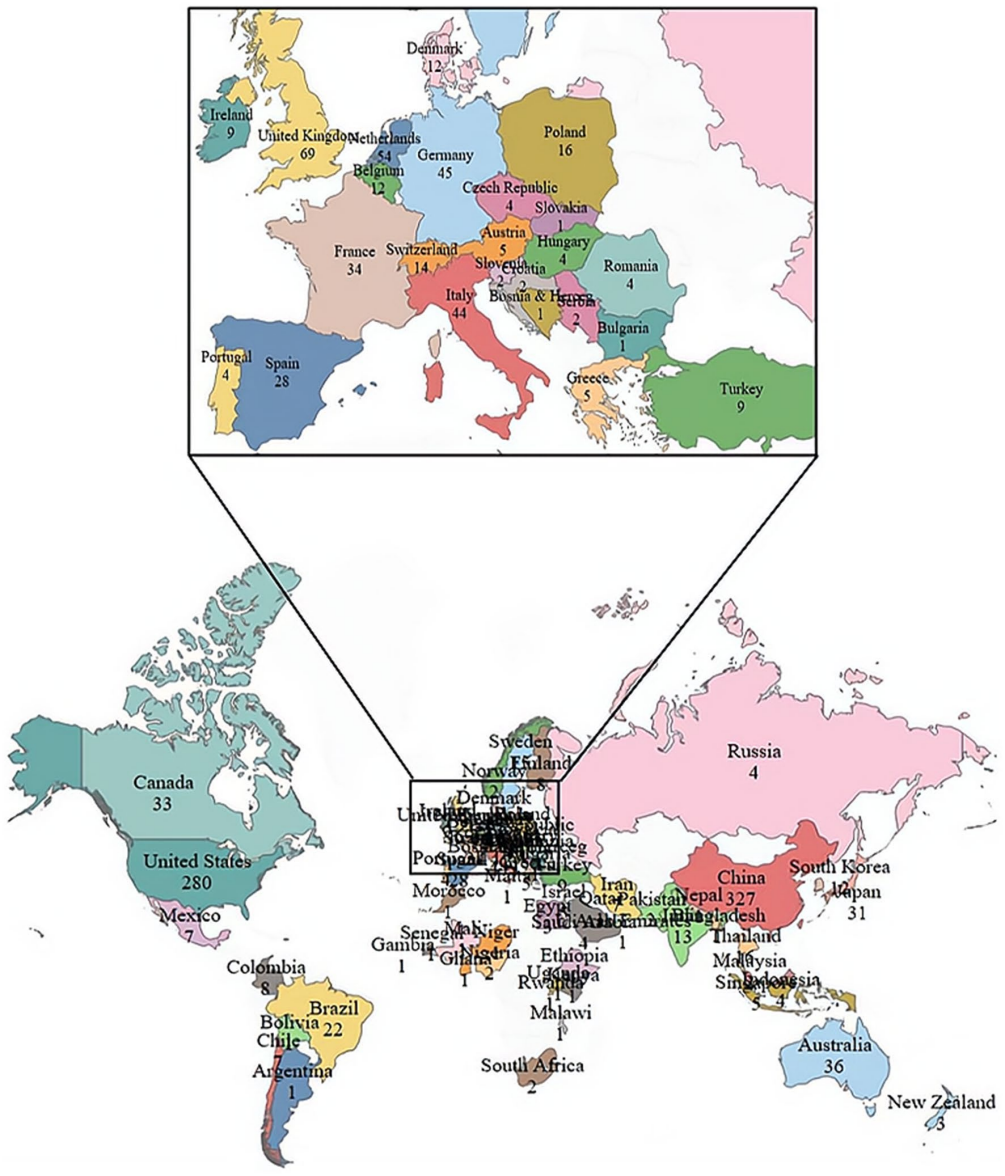
Geographic distribution of the studies on sepsis and gut microbiota.

The studies on sepsis and gut microbiota were conducted in 69 countries or regions. We screened and visualized these countries and constructed maps based on the number of publications in each country (see Figure 4). Table 3 lists the top five countries with the most publications. China topped the list with 327 entries, followed by the United States with 279. The United Kingdom, the Netherlands, and Germany ranked next. The United States had the highest total number of citations. Although China published the most articles, its average citation count per article was significantly lower than that of other countries. The Netherlands recorded the highest citations per article, which is closely linked to Professor Wiersinga, W. Joost, the most prolific author from that country (Figure 5).

**Table 3:** Top five countries in terms of the number of publications.

Rank	Country	Documents	Citations	Average citation
1	China	327	5,126	15.67
2	United States	280	13,734	49.05
3	United Kingdom	69	3,412	49.45
4	The Netherlands	54	4,389	81.28
5	Germany	45	1,112	24.71

### 3.6 Keyword analysis

#### 3.6.1 Cluster analysis of the high-frequency keywords

**Figure 6:**
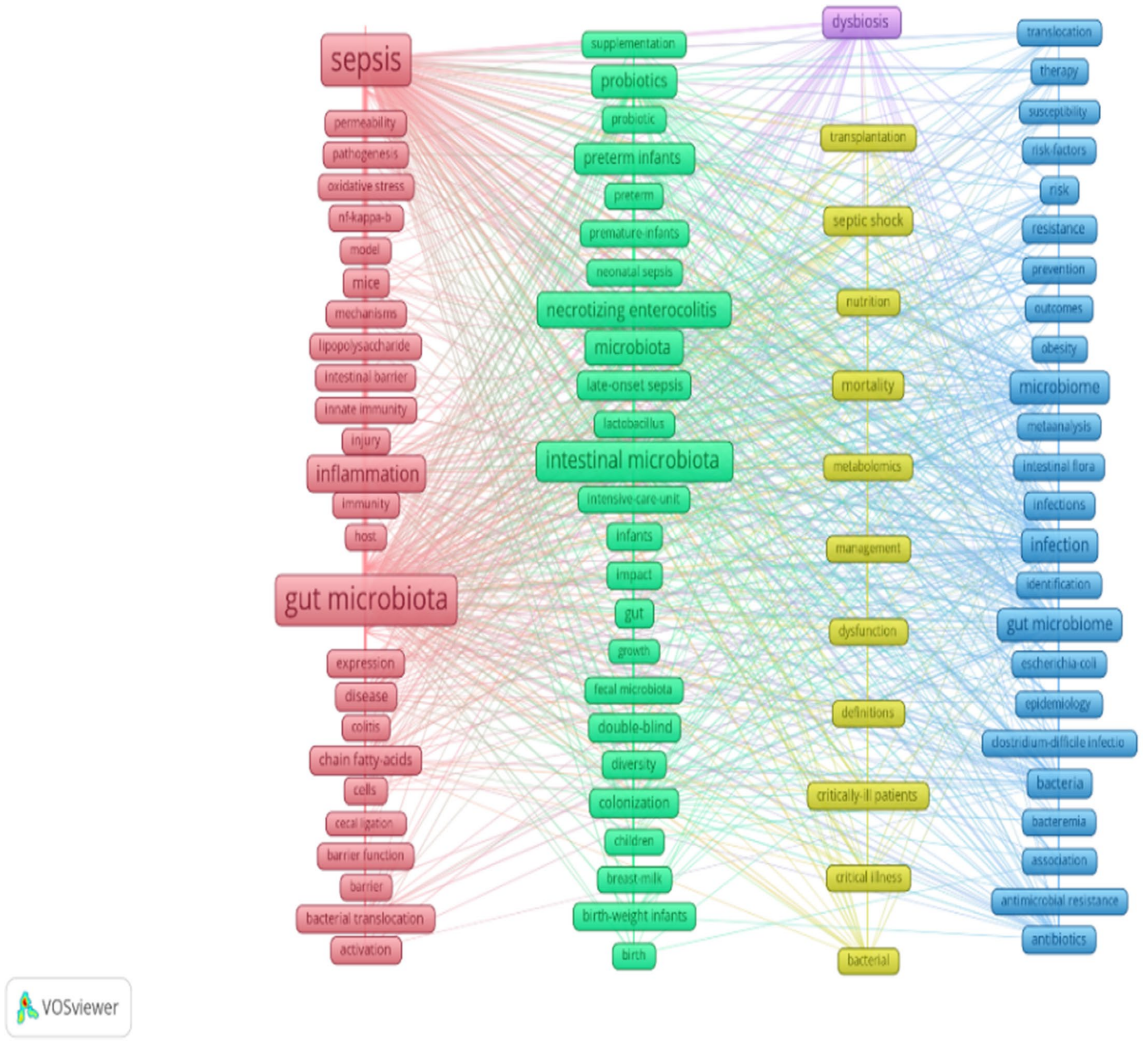
Cluster map of the high-frequency keywords.

**Figure 7:**
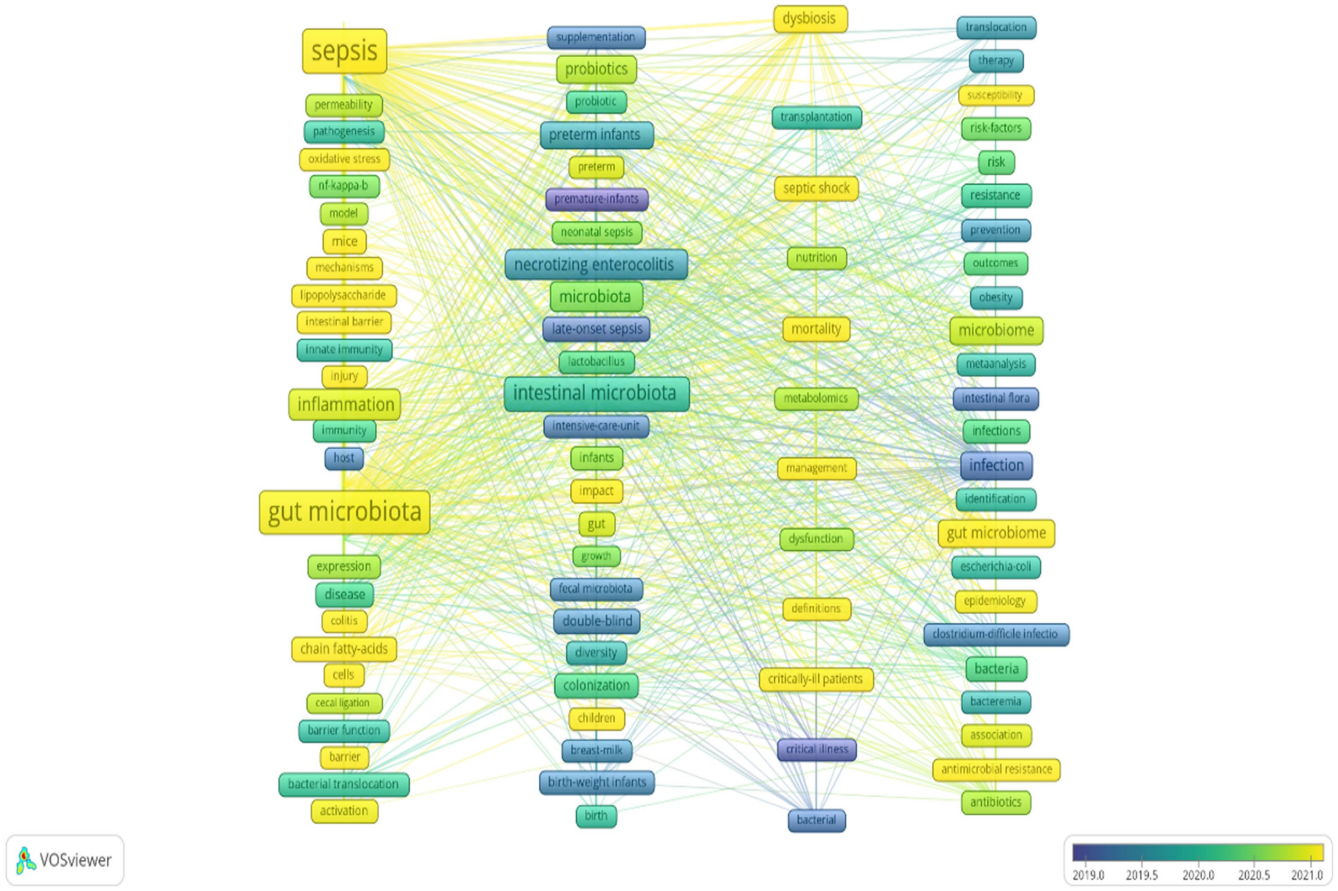
Graph of the average years of occurrence of the high-frequency keywords.

In this study, VOSviewer was used to create a keyword co-occurrence network based on the 944 studies. According to Price’s law, keywords that appeared more than 16 times were identified as core keywords. In addition to the subject terms, keywords such as inflammation, necrotizing enterocolitis, probiotics, and preterm infants also appeared frequently. The connections between the different keywords indicated that they had a co-occurrence relationship. The keywords were clustered according to the research method, and 91 core keywords were divided into five clusters. The colors of the nodes represent different clusters. Larger nodes indicate the keywords that appeared more frequently, representing key hotspots in the domain. The keywords in the red cluster in the figure were related to sepsis and its associated complications, especially the role of the gut microbiota in sepsis. The keywords in the green cluster were related to neonatal sepsis and infant health and nutrition. The keywords in the blue cluster included infection and microorganism, omics analysis, and clinical treatment and prevention. The keywords in the yellow cluster were related to the clinical management of critically ill patients, metabolomics, and nutritional support. The keyword in the purple cluster was dysbiosis, which may be related to developmental problems caused by metabolic diseases or infections. Figure 6 presents a visualization of the keyword overlay, and Figure 7 shows a visualization of the average years of occurrence of the high-frequency keywords. Keywords appearing in blue represent terms from the early stage, while those in yellow correspond to terms from the late stage, with examples of the early-stage keywords including host, fecal microbiota, double-blind, and critical illness. In recent years, keywords such as sepsis, gut microbiota, and mechanisms have been predominantly used. This indicates that research hotspots and trends have gradually shifted from basic research in the early stage to more focused studies on specific disease mechanisms and treatments. This change reflects the evolution of the understanding and research direction of sepsis and its related complications in research content.

#### 3.6.2 Keyword emergence analysis

**Figure 8:**
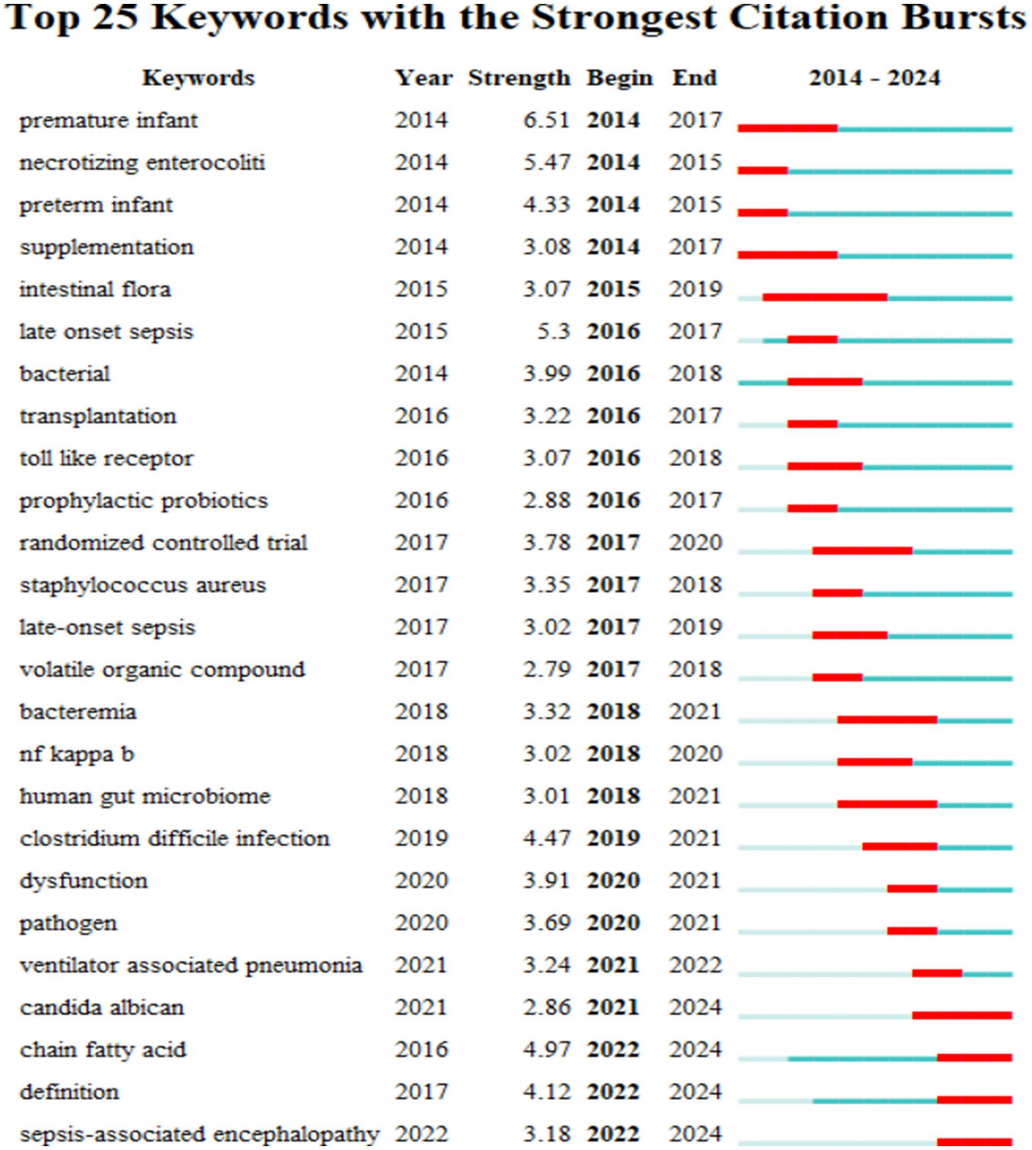
Keyword emergence map.

Keyword emergence refers to a significant increase in the frequency of a keyword within a short period of time. The CiteSpace software regards this mutation information as an indicator of transformation and evolution in research representing the emerging hotspots. The red segment in Figure 7 represents the start and end time of the emergence of the emergent keyword and the green segment represents the duration of its sustained presence. It can be seen that necrotizing enterocolitis and preterm infants were the research hotspots in the early stage of this field. Recently research on short-chain fatty acids (SCFAs) and *Candida albicans* has increased. With advances in medical treatment the mortality rate of neonatal sepsis has improved (Milton et al., 2022) and research hotspots are gradually shifting from broad topics to more detailed areas. The overall evolution of emergent keywords shows that research related to gut microbiota and sepsis continues to attract attention. Sepsis-associated encephalopathy (SAE) had a strong citation burst in 2022. It was found that increased lipocalin-2 (LCN2) led to mitochondrial dysfunction and oxidative stress

while LCN2 knockdown attenuated neuronal damage and significantly improved synaptic and cognitive impairment in septic mice (Gong et al., 2024). The expression of GDF15 was increased in the brain of septic mice (Chen et al., 2025) and targeted therapy with an anti-GDF15 antibody can improve cognitive and memory impairment caused by sepsis. These findings provide new ideas and strategies for the treatment of sepsis-associated encephalopathy (SAE) (Figure 8).

#### 3.6.3 Keyword time zone cluster analysis

The curve in the figure represents the continuous occurrence time of a keyword within a cluster, and the timeline diagram shows the most frequently appearing keywords in each cluster over time. The analysis of the timeline view of research on sepsis and intestinal microbial therapy showed that global researchers have continuously explored sepsis throughout the entire research process. Keywords for cluster #0, necrotizing enterocolitis, included “necrotizing enterocolitis,” “preterm infant,” and “intestinal microbiota,” highlighting research focused on necrotizing enterocolitis and its relationship with the intestinal microbiota. Keywords for cluster #1, intestinal barrier, included “intestinal barrier,” “bacteria,” and “transplantation.” The studies in this cluster focused on intestinal barrier dysfunction and its association with bacteria and transplantation. Keywords for cluster #2, fecal microbiota, included “fecal microbiota,” “gut microbiome,” and “microbiota.” Research in this cluster focused on the composition of fecal microbiota and its impact on health. Keywords for cluster #3, pathogen, included “pathogen,” “infections,” and “*Staphylococcus aureus*.” The studies in this cluster focused on the role and impact of pathogens in infection. Keywords for cluster #4, gut microbiome, included “gut microbiome,” “bacteremia,” and “dysbiosis.” The studies in this cluster focused on the composition and function of the gut microbiota and its relationship with sepsis. Keywords for cluster #5, gut microbiota, included “gut microbiota,” “septic shock,” and “dysfunction.” The studies in this cluster emphasized the role of the gut microbiota in sepsis and septic shock. Keywords for cluster #6, immune regulation, included “immune regulation,” “risk,” and “inflammation.” Research in this cluster focused on the role of immune regulation in gut health and disease.

**Figure 9:**
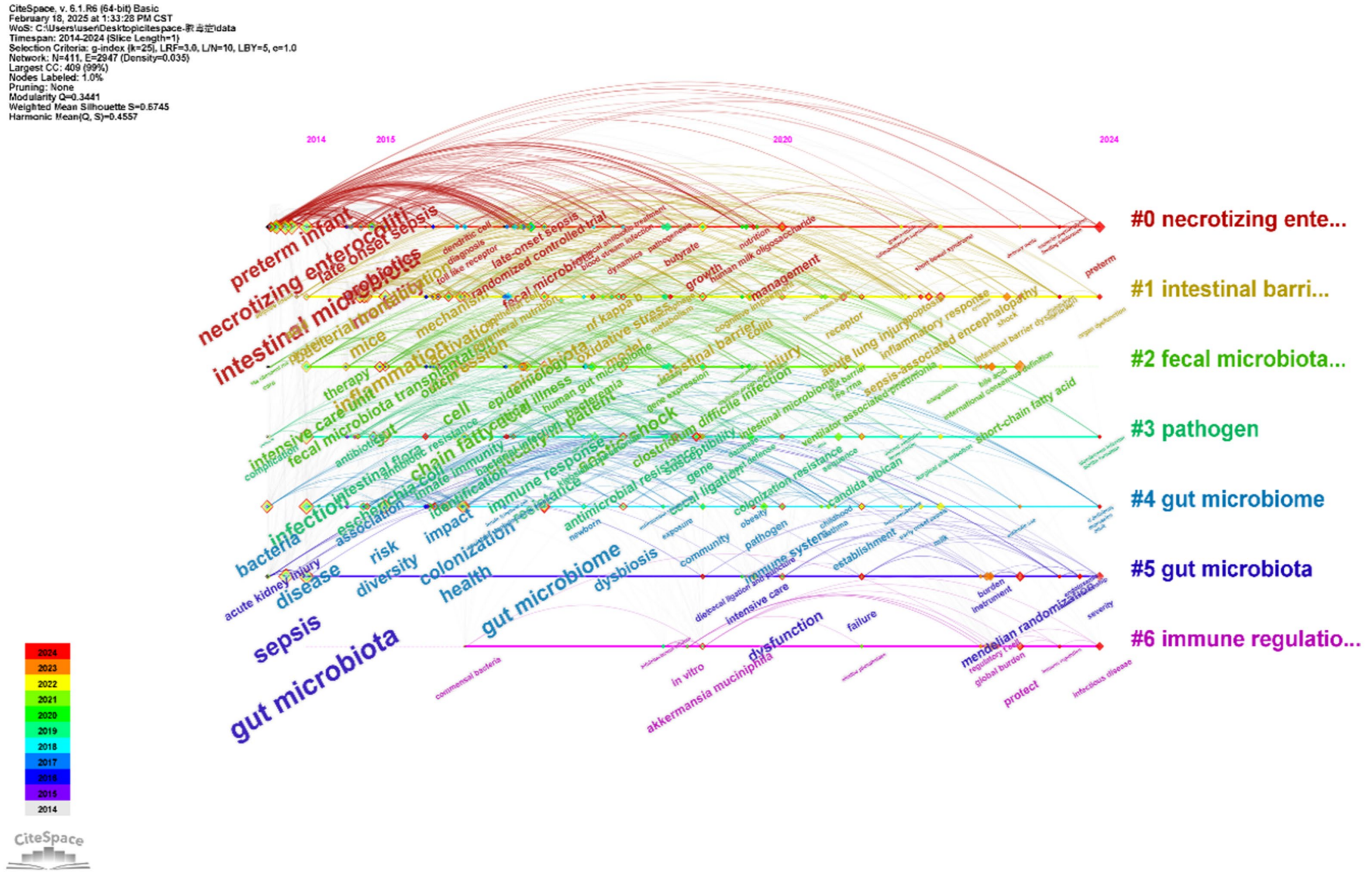
Keyword time zone cluster map.

It can be inferred from the figure that the research field forms a closed loop of “mechanism analysis-technical innovation-clinical translation.” The early research was mainly based on basic mechanisms. With the advancement of technologies such as organoids and single-cell sequencing, the field has entered a new stage. The cluster 0 studies focused on the mechanism of intestinal dysplasia and necrosis in preterm infants. In recent years, the gut microbiota and fecal microbiota have been explored as preventive means (Guo et al., 2020), representing a longitudinal shift from understanding pathological mechanisms to developing targeted interventions. The cluster 1 studies focused on the integrity and permeability of the intestinal mucosal barrier and its role in diseases, and its association with cluster 6 has become a research hotspot in recent years (Barbara et al., 2021; Singhal and Shah, 2020; Ghosh et al., 2021). At the clinical level, fecal microbiota therapy has moved from empirical treatment to evidence-based medicine. In the field of immune regulation, the fecal microbiota has emphasized the trinity network of “microbiota-immune-barrier” in recent years (Liu et al., 2020; Figure 9).

### 3.7 Co-citation analysis

#### 3.7.1 Cluster analysis of the co-cited journals

**Figure 10:**
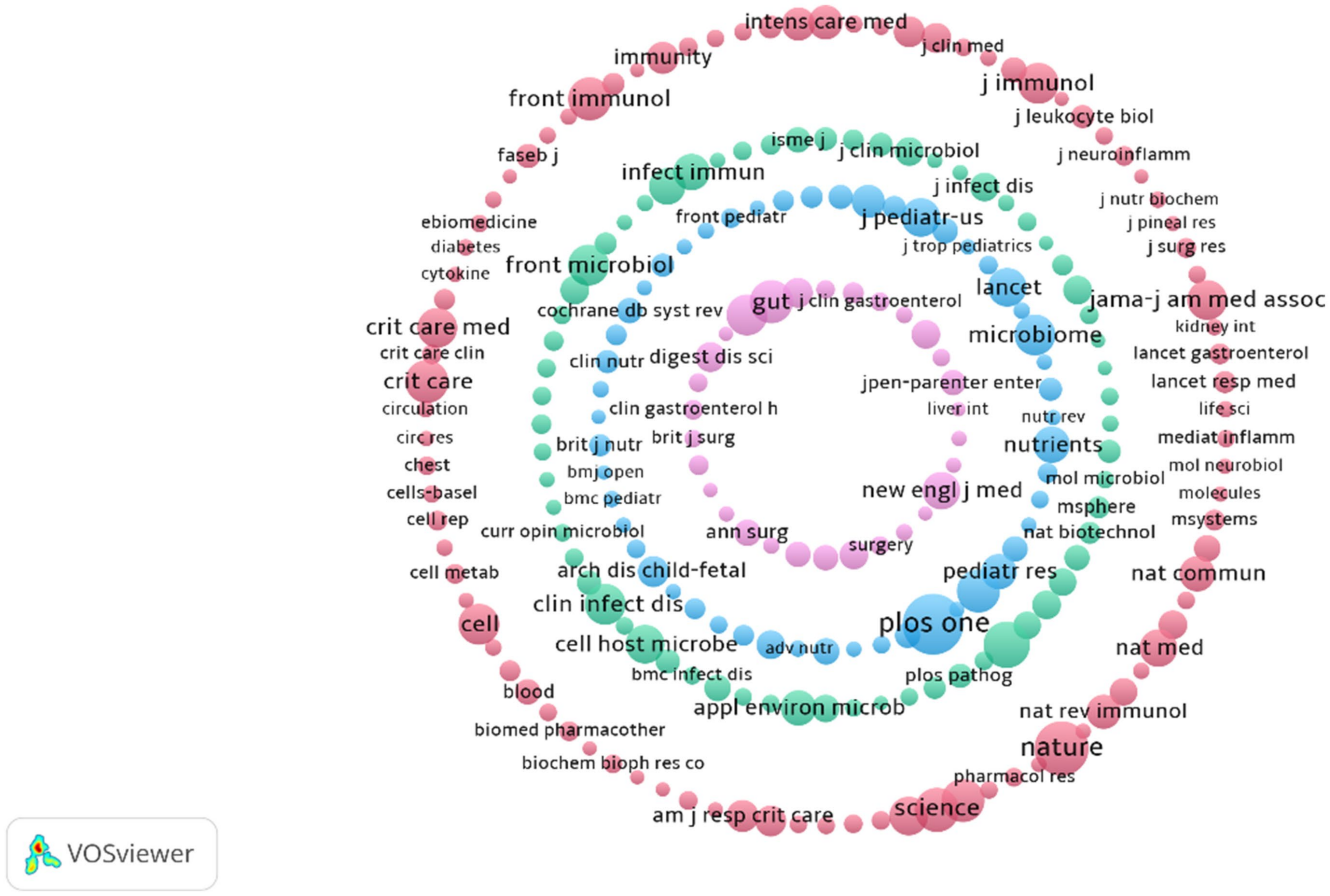
Cluster map of the co-cited journals.

Analysis of co-cited journals can identify core journals, facilitate interdisciplinary research, and explain the structure of a research domain. This study presents a visual analysis of the journals cited more than 80 times, as shown in Figure 10. The nodes in the graph represent different journals or research topics, while the size of the nodes typically corresponds to the citation frequency of the journal or topic. A total of 150 journals are divided into four clusters. The journals in the red cluster were mainly concentrated in fields such as immunology, critical care medicine, microbiology and microbiome, biomedicine and pharmacology, internal medicine, cell biology, neuroscience, and neuroinflammation. The journals in the blue cluster were mainly concentrated in fields such as pediatrics, nutrition, and perinatal medicine. The journals clustered in purple were mainly concentrated in the fields of digestive system, liver diseases, and surgery. The journals in the green cluster were mainly concentrated in the fields of microbiology, infectious diseases, immunology, biotechnology, bioinformatics, and biomedical research.

#### 3.7.2 Analysis of the co-cited references

**Figure 11:**
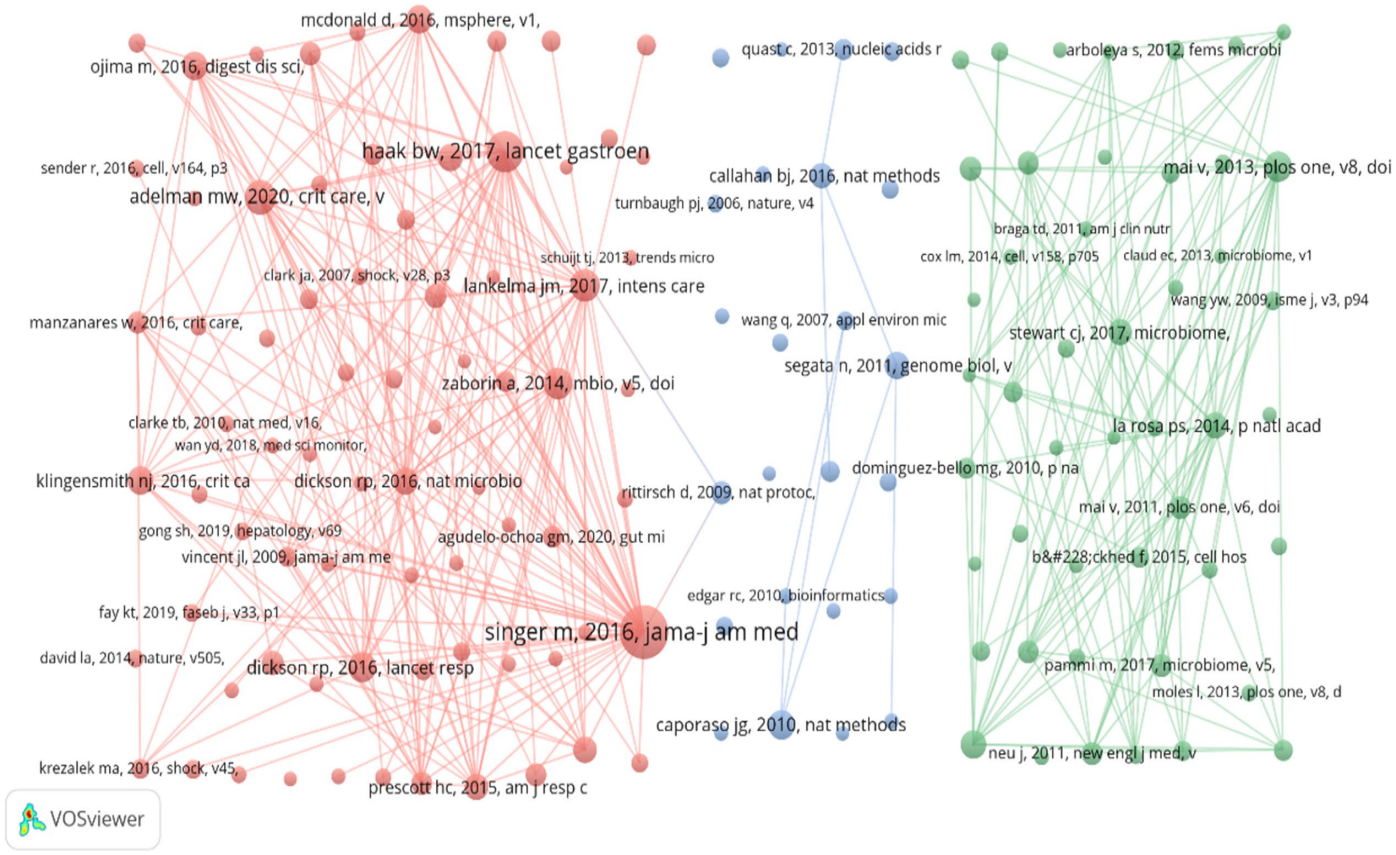
Cluster map of the highly cited literature.

Table 4 shows the top 10 most-cited articles, and Figure 11 presents a cluster visualization of the articles cited ≥20 times. The most frequently cited article was “The Third International Consensus Definitions for Sepsis and Septic Shock (Sepsis-3),” published in JAMA, which is an important international consensus. It provides updated definitions and diagnostic criteria for sepsis and septic shock (Singer et al., 2016). This consensus emphasizes that sepsis is a life-threatening organ dysfunction caused by the uncontrolled response of the body to infection, while septic shock is defined as sepsis with severe circulatory, cellular, and metabolic abnormalities that significantly increase the mortality rate. This consensus document was developed by a working group of multiple international experts to improve the accuracy of diagnosis and the effectiveness of treatment for sepsis and septic shock. The article “Enrichment of the lung microbiome with gut bacteria in sepsis and the acute respiratory distress syndrome” was published in Nature Microbiology. This article sheds light on the link between the gut and lung microbiota and their possible roles in lung diseases, such as sepsis and acute respiratory distress syndrome (ARDS). These findings provide new insights into the understanding of the pathological mechanisms underlying sepsis and ARDS and may provide new targets for future therapeutic strategies (Dickson et al., 2016). The studies “The microbiome and critical illness” and “Extreme Dysbiosis of the Microbiome in Critical Illness” highlight the relationship between microbiome dysbiosis and critical illness (Dickson, 2016). The latter study was conducted by prospectively monitoring the microbiome of ICU patients (McDonald et al., 2016). It found that critical illness results in rapid and dramatic microbiome dysregulation, characterized by depletion of beneficial bacteria and overgrowth of pathogenic bacteria. The study “Distortions in Development of Intestinal Microbiota Associated with Late Onset Sepsis in Preterm Infants” focused on the relationship between late-stage sepsis and abnormal gut microbiome development in preterm infants, revealing the role of microbiome disorders in specific populations (Mai et al., 2013). Together, these studies build a picture of microbiome dysregulation in critical illness, highlighting the critical role of the microbiome in critical illness, from the specific manifestations of microbiome dysregulation and interactions with the immune system to its impact on clinical outcomes. These studies provide a foundation for future microbiome-based diagnostic and therapeutic strategies.

**Table 4:** Top 10 cited articles.

Cited reference	Source title	Year	Cited	Doi
The third international consensus definitions for sepsis and septic shock (sepsis-3)	JAMA	2016	18,821	10.1001/jama.2016.0287
Enrichment of the lung microbiome with gut bacteria in sepsis and the acute respiratory distress syndrome	Nature Microbiology	2016	484	10.1038/nmicrobiol.2016.113
The microbiome and critical illness	Lancet Respiratory Medicine	2016	332	10.1016/s2213-2600(15)00427-0
Extreme dysbiosis of the microbiome in critical illness	Msphere	2016	292	10.1128/msphere.00199-16
Membership and behavior of ultra-low-diversity pathogen communities present in the gut of humans during prolonged critical illness	Mbio	2014	267	10.1128/mbio.01361-14
The role of the gut microbiota in sepsis	Lancet Gastroenterology and Hepatology	2017	257	10.1016/s2468-1253(16)30119-4
Distortions in development of intestinal microbiota associated with late onset sepsis in preterm infants	PLoS One	2013	203	10.1371/journal.pone.0052876
Critically ill patients demonstrate large interpersonal variation in intestinal microbiota dysregulation: a pilot study	Intensive Care Medicine	2017	199	10.1007/s00134-016-4613-z
Metagenomic analysis reveals dynamic changes of whole gut microbiota in the acute phase of intensive care unit patients	Digestive Diseases and Sciences	2016	161	10.1007/s10620-015-4011-3
Hospitalization type and subsequent severe sepsis	American Journal of Respiratory and Critical Care Medicine	2015	144	10.1164/rccm.201503-0483oc

## 4 Discussion

Sepsis is a heterogeneous disease, and traditional treatments, such as antibiotics and organ support, face many challenges in improving the prognosis of patients (Liu et al., 2017). Recent studies have shown that the gut microbiota plays an important role in the occurrence and development of sepsis. Using software tools such as CiteSpace and VOSviewer, this study analyzed research related to sepsis and intestinal flora from 2014 to 2024. It provided a visual analysis of the number of publications, publishing institutions, keywords, co-cited literature, and other aspects within this field. The map presentation enables researchers to quickly understand the hotspots and evolving trends within this research field.

Research progress indicates that future research hotspots in sepsis and gut microbiota may focus on the direct targets of the gut microbiota, the relationship between the gut microbiota and immune regulation in sepsis, the effect of the gut microbiota on the immune system in sepsis, and its potential effect on the prognosis of elderly patients with sepsis (Li et al., 2022; Vatanen et al., 2018; Zhang et al., 2022; Sun et al., 2024). Professor Ke-Xuan-Liu’s team at Southern Medical University has made significant progress in the study of the regulation of sepsis by the intestinal flora. The study showed that the novel tripeptide Arg-Lys-His (RKH) derived from the intestinal bacterium *Akkermansia muciniphila* can target TLR4 to inhibit sepsis, while phenylpyruvate secreted by the intestinal symbiont *Candida albicans* can target SIRT2 to inhibit sepsis (Deng et al., 2023). These studies will help further understand the mechanism of the intestinal commensal microbiota in regulating multiple organ damage in sepsis. They also broaden our knowledge of the interaction between the host and intestinal microbiota in the development of sepsis and provide clues and treatment ideas for understanding the “microbiota–host” interaction in sepsis.

The theoretical mechanisms and therapeutic approaches derived from gut microbiota research in sepsis have far-reaching implications beyond clinical medicine. Microbial communities possess complex metabolic capabilities, particularly in breaking down complex substrates and synthesizing bioactive compounds, making them ideal candidates for fields such as biofuel production, green chemistry, and environmental protection. For example, gut-derived microbes and their enzymes have been utilized in biofuel production (Ao et al., 2024). Similarly, microbial metabolites such as short-chain fatty acids (SCFAs), which play a pivotal role in immune regulation during sepsis, also hold significant potential for applications in green chemistry. Furthermore, research on the interactions between surfactants and biofilm formation in *Staphylococcus aureus* and *Candida albicans* species provides novel insights for developing innovative anti-biofilm strategies (Aonofriesei, 2024), Recent studies have enhanced microbial community stability through interspecies interactions, drawing inspiration from host–microbiota interplay mechanisms uncovered in sepsis research to improve stress adaptability in industrial microbial consortia (Zhang et al., 2023) In the field of bioenergy, chitinases have shown unique value by efficiently converting chitin from crustacean waste into N-acetylglucosamine, which can then be fermented to produce bioethanol (Poria et al., 2024).

This study offers several methodological advancements compared to similar research in the field, employing a more diverse set of visualization tools to clarify the knowledge flow from molecular biology to clinical medicine while dynamically illustrating the shift in research hotspots from “necrotizing enterocolitis” to “short-chain fatty acids.” In addition, we propose a closed-loop research framework encompassing “mechanistic analysis → technological innovation → clinical translation.” However, affected by some objective factors, this study also has some limitations. Firstly, bibliometric analysis software has high specifications and standards for data. To ensure the quality and integrity of the collected data, this study only selected English-language publications in the SCIE index of the core collection of the Web of Science database, excluding other databases and non-English literature. This selection criterion might have resulted in potential insufficiencies in the data analysis. Secondly, this study only included literature from 2014 to 2024, which might have missed important research and findings published prior to 2014. In addition, quantitative analysis requires interpretation of the data, which inevitably introduces some degree of subjectivity. It is recommended that future studies incorporate long-term follow-up and provide a more comprehensive discussion of the research methods and conclusions presented in the literature.

## Data Availability

The original contributions presented in the study are included in the article/supplementary material, further inquiries can be directed to the corresponding authors.

## Footnotes

^1^ https://donghaoren.org/charticulator/
